# Apolipoproteine and *KLOTHO* Gene Variants Do Not Affect the Penetrance of Fragile X-Associated Tremor/Ataxia Syndrome

**DOI:** 10.3390/ijms25158103

**Published:** 2024-07-25

**Authors:** Tri Indah Winarni, Ye Hyun Hwang, Susan M. Rivera, David Hessl, Blythe P. Durbin-Johnson, Agustini Utari, Randi Hagerman, Flora Tassone

**Affiliations:** 1 Center for Biomedical Research (CEBIOR), Faculty of Medicine, Universitas Diponegoro, Semarang 50275, Central Java, Indonesia; triindahw@gmail.com (T.I.W.); agustiniutari@gmail.com (A.U.); 2 Department of Biochemistry and Molecular Medicine, School of Medicine, University of California Davis, Sacramento, CA 95817, USA; yehhwang@ucdavis.edu; 3 Department of Psychology, University of Marlyand, College Park, MD 20742, USA; srivera@ucdavis.edu; 4 MIND Institute, University of California Davis Medical Center, Sacramento, CA 95817, USA; drhessl@ucdavis.edu (D.H.); rjhagerman@ucdavis.edu (R.H.); 5 Department of Psychiatry and Behavioral Sciences, School of Medicine, University of California Davis, Sacramento, CA 95817, USA; 6 Division of Biostatistics, School of Medicine, University of California Davis, Davis, CA 95616, USA; bpdurbin@ucdavis.edu; 7 Department of Pediatrics, Faculty of Medicine, Universitas Diponegoro, Semarang 50275, Central Java, Indonesia; 8 Department of Pediatrics, School of Medicine, University of California Davis, Sacramento, CA 95817, USA

**Keywords:** *FMR1* gene, FXTAS, premutation, *KLOTHO*, *APOε4*

## Abstract

In this study, the potential role and interaction of the *APOε* and *KLOTHO* genes on the penetrance of fragile X-associated tremor/ataxia syndrome (FXTAS) and on the IQ trajectory were investigated. FXTAS was diagnosed based on molecular, clinical and radiological criteria. Males with the premutation (PM) over 50 years, 165 with and 34 without an FXTAS diagnosis, were included in this study and were compared based on their *APO* (*ε2-ε3-ε4*) and *KLOTHO* variant (*KL-VS*) genotypes. The effect of *APOε4* on FXTAS stage and on diagnosis did not differ significantly by KL-VS genotype with interaction effect *p* = 0.662 and *p* = 0.91, respectively. In the FXTAS individuals with an *APOε2* allele, a marginal significance was observed towards a larger decline in verbal IQ (VIQ) in individuals with an *APOε4* allele compared to those without an *APOε4* allele (*p* = 0.071). In conclusion, our findings suggest that the *APOε4* and *KL-VS* genotypes alone or through their interaction effect do not appear to predispose to either FXTAS diagnosis or stage in male carriers of the PM allele. A further study is needed to establish the trend of IQ decline in the FXTAS individuals who carry *APOε4* with *APOε2* compared to those without *APOε4*.

## 1. Introduction

Carriers of the fragile X messenger ribonucleoprotein 1 (*FMR1*) premutation (PM) allele (55-200 CGG repeats) are at increased risk of developing fragile X premutation-associated conditions (FXPAC) [1]. These include fragile X-associated primary ovarian insufficiency (FXPOI), fragile X-associated tremor/ataxia syndrome (FXTAS), fragile X-associated neuropsychiatric disorders (FXAND), and a number of fragile X-associated conditions such as immune-mediated disorders, sleep apnea, hypertension, and migraine [1,2].

FXTAS is a late-onset progressive neurodegenerative disorder, characterized by intention tremor, gait ataxia, difficulty with ambulation, deficits in executive function, and brain atrophy. The penetrance in males is 47–75%, and the highest penetrance is elderly male carriers. The penetrance in female carriers is lower at 16% [3,4].

The apolipoprotein E (*APOε*) is a component of lipoprotein complexes that has a multifunctional role in the homeostasis of cholesterol, neurobiology, and in neurodegenerative diseases. *The APOε* gene, located on the 9q13.3 chromosome, has three major isoforms: *APOε2*, *APOε3*, and *APOε4* alleles, and it is associated with cognitive impairments particularly in Alzheimer’s disease (AD) [5]. The *APOε2* allele has been shown to be protective in AD against amyloid (Aβ) accumulation, which is characterized by the deposition of the abnormal amyloid protein in the regions of the brain, associated with cognitive impairment in the early stages of AD [6]. The *APOε3* allele, the most common in the general population, is neutral, while the *APOε4* allele represents the strongest genetic risk factor for AD in all ethnic groups [5]. It acts in a gene dose-dependent manner increasing the risk by up to 15-fold in homozygotes across all ages and sex between 40 and 90 years [7,8]. Further, the increased risk associated with the *APOε4* allele appears to be more dominant than the protective effect observed for the *APOε2* allele [9].

The prevalence of the *APOε4* allele is 13.7% in the general population [10]; however, it ranges from 9% to 23% in diverse ethnic populations [10,11]. In those with mild cognitive impairment and AD, the prevalence of *APOε4* dramatically increases to 36% and 42%, respectively [11,12].

On the contrary, the *KLOTHO* gene, located at 13q13, encodes for a transmembrane protein that has been associated with enhanced longevity and better brain health in aging. The gene is believed to act as a putative age-suppressing gene in wild-type mice, where, when overexpressed, it extends their life span and when disrupted, induces complex phenotypes of human premature aging syndromes [13]. Loss-of-function mutations in mice are associated with reduced *KLOTHO* protein expression and accelerated aging phenotypes [14]. In humans, *KLOTHO* gene heterozygosity (*KL-VS^het^*) leads to increased blood protein levels and protection from cognitive impairments [15]. In a recent study, the combined effect of the *APOε4* allele and *KLOTHO* variants in a large longitudinal AD cohort was explored, and the protective effect of *KLOTHO* variants (*KL-VS*) was powerfully demonstrated against the *APOε4* allele by reducing the risk of converting healthy patients carrying the *APOε4* allele from aging to mild cognitive impairment (MCI). Also, patients carrying the *KL-VS^het+^* genotype and the *APOε4* allele were protected from the conversion of MCI to AD [16].

Interestingly, a high allele frequency (36.3%) of *APOε4* was reported among *FMR1* PM individuals as a potential predisposing factor and associated with the early onset of FXTAS with progressive cognitive decline [17]. Fifty percent of men with FXTAS eventually become cognitively impaired [18,19]; specifically, cognitive and executive impairments develop about four years after the onset of motor symptoms and worsen with FXTAS stages. In a study, it was suggested that executive impairment is more prominent than memory loss in FXTAS [20]. Unlike dementia in AD, multiple cognitive impairments (executive function, memory retrieval and recall, attention, receptive and expressive language, and visuospatial skills) and psychomotor slowing, which reflect mixed cortical–subcortical dementia, are prominent in FXTAS. They involve both cortical (hippocampal, frontal) and subcortical (middle cerebellar peduncles) areas, while memory impairment, aphasia, and apraxia reflect cortical dementia observed in AD [19,21].

The present study aimed to investigate the allele frequency of *APOε* and *KLOTHO* gene variants (*KL-VS*) in 245 premutation males over 50 years with and without FXTAS and to investigate the potential role of the allelic variants of these two genes in the pathogenesis of FXTAS, as well their role in the cognitive decline observed in aging premutation carriers.

## 2. Results

In total, the frequency of *APOε* and *KLOTHO* alleles were determined in 245 male carriers, including 165 with the FXTAS diagnosis, 34 without an FXTAS diagnosis, and 46 individuals whose data on FXTAS diagnosis were unavailable. The mean age was 66.1 (±7.93), and the mean of CGG repeat was 86.9 (±18.8) with no AGG interruptions in nearly half of the participants (49.8%, Table 1). The mean age of PM with FXTAS was 66.2 (±7.44) and PM without FXTAS was 63.3 (±6.40) years, with FXTAS subjects significantly older (*p* = 0.0198) The number of CGG repeats in the FXTAS group was significantly higher (*p* = 0.0005) compared to the non-FXTAS group, at 89.4 (±18.6) and 77.4 (±17.0), respectively. The majority of PM individuals with and without FXTAS had no AGG interruptions, followed by one AGG interruption with a similar percentage, 30.3% in individuals with FXTAS and 31.4% in individuals without FXTAS. The presence of two AGG interruptions was more frequent in PM individuals who had FXTAS (19.4%) compared to those who did not have FXTAS (11.4%) (Table 2). However, the number of participants in the non-FXTAS group was five times lower (*n* = 34) than in the group of participants with the diagnosis of FXTAS (*n* = 165).

Individuals with the *APOε3,ε3* genotype were the majority (66.5%), followed by the *APOε3,ε4* genotype observed in 19.2% of the participants. Only 0.4% of carriers of the PM had two copies of the *APOε4* allele while 21.6% had one copy; thus, in total, the frequency of the *APOε4* allele was 20.6% and 17.1% in participants with and without FXTAS, respectively, which was not significantly different (*p* = 0.713). Non-Hispanic white formed the majority of the participant ethnicity in this study (73.5%) and 22.5% were unknown; thus, the effect of the *APOε* and *KLOTHO* variants on the penetrance of FXTAS could not be assessed by ethnicity (Table 2).

The *KL-VS^het−^* allele was observed in the majority of participants (67.8%), 28.2% was the frequency of *KL-VS^het+^*, and only 4.1% of participants had two copies of the *KL-^hom+^* variant; therefore, the total frequency of the *KL_VS* allele was 32.3%. Among participants who had FXTAS, the frequency of *KL-VS^het+^* was 30.3%, and 28.6% was the frequency observed in those without FXTAS. Three percent of participants with FXTAS carried *KL-^hom+^* and none was found in those without FXTAS (Table 2). Further, 26.5% of the participants were diagnosed with FXTAS stage 3, followed by stage 2 (17.6%), and stage 4 (15.9%). A definite diagnosis of FXTAS was determined in ~40% of cases (Table 1).

### 2.1. FXTAS Stage by APOε4 and KLOTHO Variant Genotypes

Table 3 shows the results of the proportional odds logistic regression model of the FXTAS stage (as an ordered categorical variable) by *APOε4* and KL-VS genotypes. The odds of a higher vs. lower FXTAS stage did not differ significantly by *APOε4* genotype for either KL-VS category, and did not differ significantly by KL-VS genotype for either *APOε4* genotype category. Furthermore, the effect of *APOε4* on FXTAS stage did not differ significantly by KL-VS genotype (interaction effect *p* = 0.662). The stacked bar plot of FXTAS stage by *APOε4* and KL-VS can be seen in the Supplementary Materials, Figure S1. FXTAS stage frequencies were found to be similar in two bar plots of no *APOε4* allele presentation; however, in individuals who had *APOε4* allele, a higher FXTAS stage (FXTAS stage 3, 4, and 5) was observed in individuals with KL-VS (KL-VS^het+^ + or KL-VS^hom+^) compared to those without KL-VS, but the difference did not reach statistical significance.

### 2.2. FXTAS Diagnosis by APOε4 and KLOTHO Variant Genotype

Table 4 shows the results of the proportional odds logistic regression model of FXTAS diagnosis (as an ordered categorical variable) by *APOε4* and *KLOTHO* genotypes. The odds of a higher vs. lower FXTAS diagnosis did not differ significantly by *APOε4* genotype for either *KLOTHO* genotype category, and did not differ significantly by *KLOTHO* genotype for either *APOε4* genotype category. Moreover, the effect of *APOε4* on FXTAS diagnosis did not differ significantly by *KLOTHO* genotype (interaction effect *p* = 0.91). The stacked bar plot of FXTAS diagnosis by *APOε4* and KL-VS can be seen in Figure S2. The frequency of FXTAS diagnosis was similar, as shown in the two bar plots of no *APOε4* allele presentation; however, in individuals who had *APOε4* allele, the FXTAS diagnosis was more confident (only possible, probable, and definite) in those individuals with KL-VS (KL-VS^het+^ + or KL-VS^hom+^) compared to those without KL-VS (KL-VS^het+^ + or KLVS-^hom+^).

### 2.3. FXTAS Stage and Diagnosis by APOε2 and ε4 Alleles

Table 5 shows the results of the proportional odds logistic regression model of FXTAS stage (as an ordered categorical variable) by *APOε2* and *ε4* genotypes. The odds of a higher vs. lower FXTAS stage did not differ significantly by the presence of an *APOε4* allele regardless of *APOε2* genotype, and vice versa. Furthermore, the effect of *APOε4* on FXTAS stage did not differ significantly by *APOε2* (interaction effect *p* = 0.965). The stacked bar plots of FXTAS Stage by *APOε2* and *ε4* genotypes are shown in Figure S3. Similarly to the FXTAS diagnosis, the FXTAS stage frequency did not differ between the two bar plots of no *APOε4* allele presentation; however, in individuals who had *APOε4* allele, the FXTAS stage was higher (FXTAS stage 3, 4, and 5) in individuals with the *APOε2* allele compared to those without.

Table 6 shows the results of the proportional odds logistic regression model of FXTAS diagnosis (as an ordered categorical variable) by *APOε2* and *APOε4* genotypes. The odds of a higher vs. lower FXTAS diagnosis did not differ significantly by *APOε4* regardless of *APOε2* genotype and vice versa. Furthermore, the effect of *APOε4* on FXTAS diagnosis did not differ significantly by *APOε2* (interaction effect *p* = 0.906). Stacked bar plot of FXTAS diagnosis by *APOε2* and *ε4* genotypes are shown in Figure S4. FXTAS diagnosis frequencies were similar in the two bar plots of no *APOε4* allele presentation; however, in the individuals who had an *APOε4* allele, the FXTAS diagnosis was more confident (only probable, and definite) in individuals with *APOε2* compared to those without *APOε2*.

### 2.4. Changes Overtime in VIQ by APOε2 and ε4 Among FXTAS Subjects

**Figure 1:**
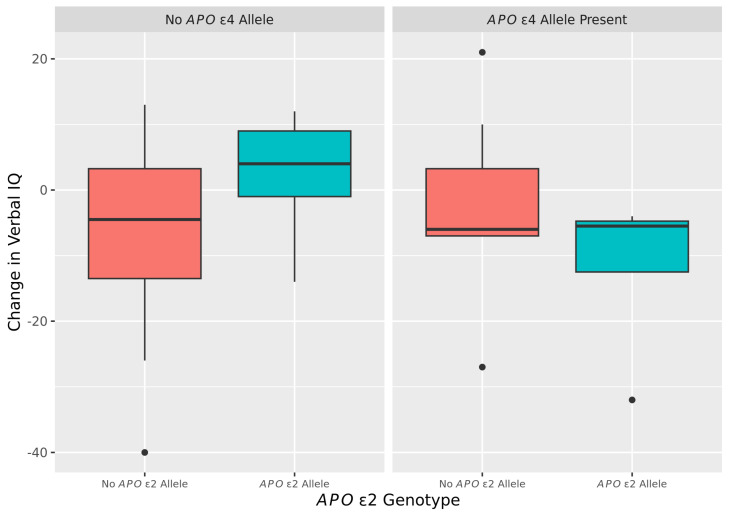
Boxplots of change in verbal IQ by *APOε2* and *ε4* alleles among FXTAS subjects (*n* = 65).

In a subgroup of participants (65 with FXTAS) for whom IQ data were available at multiple visits, further statistical analysis was performed to investigate the role of *APOε2* and *ε4* alleles on IQ (VIQ, PIQ, and PIQ) changes over time. Table 7 compares changes in VIQ between first and subsequent visits (1–6 years apart) among FXTAS subjects with (*n* = 14) and without an *APOε2* allele (*n* = 51), by *APOε4*, adjusting for the time between visits, age at first visit, and VIQ at first visit. The change in VIQ did not differ significantly by the presence of the *APOε2* allele, regardless of the *APOε4* status. However, for subjects with an *APOε2* allele present, a trend was seen towards a larger decline in VIQ in subjects with an *APOε4* allele (*n* = 12) compared to those without an *APOε4* allele (*n*= 53) with *p* = 0.071. The box plots of change in VIQ by *APOε2* and *ε4* alleles are shown in Figure 1.

### 2.5. No Changes Overtime in PIQ and FSIQ by APOε2 and ε4 Alleles among FXTAS Subjects

The comparison of the change in both FSIQ and PIQ between the first and subsequent visit between FXTAS subjects with and without an *APOε2* allele (*n* = 14, *n* = 51, respectively), by *APOε4*, did not differ significantly by the presence of an *APOε2* allele, regardless of *APOε4* status. The comparison of the change in FSIQ and PIQ between first and subsequent visits (1–6 years apart) among FXTAS subjects with and without an *APOε4* allele (*n* = 12, *n* = 53, respectively), by *APOε2*, did not differ significantly by the presence of an *APOε4* allele, regardless of *APOε2* status.

## 3. Discussion

The presence of the *APOε4* allele is strongly associated with a greater risk of cognitive impairments including dementia [6]. The prevalence of the *APOε4* allele is 13.7% in the general population [10], and it dramatically increases among those with mild cognitive impairment and AD with a frequency of 36% and 42%, respectively [12]. In this study, the frequency of the *APOε4* allele was detected in 22% of the males with a PM allele. Compared to the general population, the frequency of *APOε4* in PM males is then significantly increased (*p* = 0.0003). A similar frequency of the *APOε4* allele was observed in our participants with and without FXTAS, 20.6% and 17.1%, respectively. Interestingly, although we detected a higher frequency of the *APOε4* allele than in the general population, it was much lower than the one from a previous reported study in Spain (Silva et al., 2013). In the study, involving 44 males (mean age 70.1 years with and 71 years without FXTAS) and females (mean age 61.6 years with and 55.1 years without FXTAS) with a PM allele (22 with FXTAS, 22 without FXTAS) a high prevalence of the *APOε4* allele was identified (overall 36.3%, 31.8% in males, and 4.5% in females) similar to the prevalence in the population with mild cognitive impairment, which is more than 30% [17,22].

Carrying one copy, but not two copies of *KL-VS* [23], referred to as *KL-VS* heterozygosity (*KL-VS^het^*) leads to the overexpression and increase in the *KLOTHO* expression levels in the blood and to an increased clearance of Aβ accumulation in the brain decreasing the risk of dementia in those individuals with AD who also carry the *APOε4* allele [24]. The potential role of the allelic variants of these two genes in the pathogenesis of FXTAS has not been clearly addressed. The findings of this study do not support the role of these two genes in FXTAS, regardless of the *APOε* or the *KLOTHO* genotype.

The potential effect of *APOε4* allele on FXTAS identified in our study is not in agreement with a previous study in which it was found that the *APOε4* allele is a genetic susceptibility in PM, in both 44 males (*n* = 21) and females (*n* = 23) for a higher risk of developing FXTAS [17]. This could be due to the small sample size included in the study; having a correct sample size is critical to ensure sufficient power, able to extrapolate the statistical analysis results to the overall population [25]. The evidence generated from small sample sizes may lead to error, both false negatives due to inadequate power and false positives due to biased samples; therefore, the degree of evidence needs further study with appropriate sample sizes to assure replicability and generalizability [26]. Furthermore, it was identified in previous studies that the main known risk factors of FXTAS are age and sex; by the 80s, more than 75% premutation males will develop FXTAS [3], but females are less likely to develop FXTAS compared to males [27].

Cognitive impairment, a minor clinical criterion of FXTAS, includes moderate short-term memory deficits and executive function deficits [28]. In FXTAS, the cognitive impairments were observed in approximately 50% from mild to significant conditions [29] compared to older adults in the general population, where cognitive impairments are observed in 22% [30]. Importantly the pathogenesis of general cognitive impairments and FXTAS are quite different and may also be mixed because age is the main factor of penetrance for both conditions. An age-related dysfunction of energy metabolism leading to neurodegeneration and massive death of neuronal cells and a progressive involution of the brain in aging reflects reduced cognitive and motor activity [31]. In addition, intracellular accumulation of hyperphosphorylated tau and extracellular deposition of amyloid beta (Aβ) protein may result in cognitive impairment [32]. In FXTAS, besides age and sex, the main factor or major neuropathological criteria of FXTAS is the presence of neuronal intranuclear inclusion due to elevated level of *FMR1* mRNA and neuronal toxicity led by the CGG repeat number [33]. Previous studies have reported that increasing CGG repeats length may be a risk factor for FXTAS [34,35,36]. A study involving families with FXS showed that a mid to large CGG repeat size (70–200 CGG) significantly associated with the penetrance (6-fold increased risk) of cognitive impairment compared to small size (55–67 CGG), besides educational level and age [37]. In this study, the CGG repeats length was significantly higher in participants who had FXTAS compared to those who did not has FXTAS.

There are a variety of factors that may affect the development of cognitive impairment and FXTAS progression [38]. The stages of FXTAS correlate with the severity of involvement including progressive cognitive impairment [39]. Cognitive impairment in older adults has been widely studied especially the association between depression and the onset of dementia. In a large 14-year longitudinal study involving almost 5000 healthy males over 70 years, it was demonstrated that the males who had a history of depression were at higher risk of developing dementia [40]. Psychiatric disorders such as depression (65%) or anxiety (52%) in FXTAS can worsen cognitive decline and should be treated to prevent the progression of diseases [41,42]. Cardiovascular medical conditions, such as diabetes mellitus, hypertension and hypothyroidism, which were found to be higher in PM carriers also heighten the risk of cognitive impairment and the progression of FXTAS. Also, the progression of FXTAS may be associated with cerebrovascular diseases, a major contributor to later-life dementia, accounting for up to 20% of cases of dementia [43,44,45]. Further analysis was performed to investigate the role of *APOε2* and *ε4* alleles in IQ (VIQ, PIQ, and PIQ) changes. The IQ (full scale, verbal, and performance) scores were compared between *APOε* genotypes using linear models for *APOε4* allele presence/absence, *APOε2* allele presence/absence, the *APOε2*-*APOε4* interaction, age at first visit, time between visits, and IQ score at first visit. This study showed that in VIQ, FSIQ, and PIQ, the changes did not differ significantly by the presence of an *APOε4* allele, regardless of *APOε2* status. However, for subjects with an *APOε2* allele, a trend was seen towards a larger decline in verbal IQ in subjects with an *APOε4* allele compared to those without an *APOε4* allele. APOE gene encodes an apolipoprotein E, which when combined with fats becomes a major cholesterol carrier that supports lipid transport and repair in the brain, called lipoproteins. There are three major alleles, i.e., *APOε2*, *APOε3*, and *APOε4*, which have a frequency of 8.4%, 77.9%, and 13.7%, respectively. The *APOε3* is the most common genotype associated with normal lipid metabolism. The *APOε4* allele is the most significant genetic risk factor of CI and AD due to an imbalance between the production and clearance of amyloid-β (Aβ) peptides [7,46], the frequency dramatically increases to approximately 40% in AD population [10]. Meanwhile, the *APOε2* allele is suggested as protective for CI, and carrying it is associated with a slower rate of cognitive decline [47]. In this study, the presence of the *APOε2* allele in individuals who carry *APOε4* might cause a protective effect against VIQ declines. Further study is warranted, including a larger cohort of subjects with FXTAS, to explore more closely a plausible explanation of this phenomenon.

Based on the neuropathological mechanisms, there is a different underlying neuropathological mechanism between AD and FXTAS. The brain involvement, and the symptoms, of AD characterized by cortical/fronto-temporal dementia [48] with early signs are changes in memory, thinking, and reasoning skills, with progression to severe symptoms comprising signs such as confusion, changes in behavior, and impaired personality that interferes with social and working skills (Goedert et al., 2012). Instead, a fronto-subcortical dementia is dominant in FXTAS [49] involving the white matter and the middle cerebellar peduncle, characterized by cognitive slowing, retrieval deficits but preserved recall, dysexecutive functioning, lack of initiative, personality changes (apathy, disinhibition), and mood disturbances, accompanied by pyramidal and extrapyramidal signs such as tremor and ataxia [19,50,51].

Study Limitation. There are a limited number of participants who had multiple IQ sets assessed at visits and a wide variability in the time period between visits, which may be the reason why IQ changes are not be strongly associated with the *APOε4* in individuals with FXTAS.

In conclusion, the allele frequency of *APOε4* is 22% in PM males over 50 years, a similar frequency is found in those who have FXTAS (20.6%), and it is slightly lower in those who have no FXTAS (17.6%). There is no effect of the *APOε4* allele or the interaction effect of *APOε4* by *KLOTHO* genotypes on FXTAS diagnosis and stage. The presence of the *ε2* allele, known as a protector, is not supported in this study; however, individuals who carry the *APOε2* allele show a trend of a larger decline in verbal IQ in those with an *APOε4* allele compared to those without an *APOε4* allele. This may due to the small number of patients and limited data (*n* = 65)

## 4. Materials and Methods

### 4.1. Participants

Participants were male carriers of a PM whose CGG repeat allele size (CGG 55–200) was confirmed by both Southern blot and PCR approaches. A total of 245 males over 50 years of age and with a PM allele were included in this study. A clinical assessment for FXTAS diagnosis was available for a subset of 199 participants, including 165 individuals with FXTAS and 34 individuals without FXTAS. Besides FXTAS diagnosis and stage, IQ score was measured in 73 participants (65 with FXTAS and 8 without FXTAS). For a subset of verbal IQ (VIQ), performance IQ (PIQ), and full-scale IQ (FSIQ) in the first visits and subsequent visits for individuals who were diagnosed with FXTAS, periods for the first and subsequent visits ranged between one to six years.

All participants were recruited from the Fragile X treatment and Research Center, at the MIND Institute at the University of California, Davis, CA, USA. Individuals who received a diagnosis of FXTAS were assigned as a case and those who did not were assigned as a carrier of a PM.

This study and all research protocols were carried out in accordance with the Institutional Review Board (IRB) at the University of California, Davis with written informed consent obtained from all participants in accordance with the Declaration of Helsinki.

### 4.2. Molecular Measures

Genomic DNA (gDNA) was isolated from 3 mL of peripheral blood samples collected from 245 participants included in this study using the Gentra Puregene Blood Kit (Qiagen, Valencia, CA, USA) and was utilized for measuring the *FMR1* CGG allele size, number of the AGG interruptions, and genotyping (*APOε* and *KLOTHO*). CGG repeat number and number of AGG interruptions were determined using PCR as previously described [52,53,54]. Capillary electrophoresis (CE) was used for the visualization and sizing of the PCR products and Peak Scanner Software 2.0 for the analysis. *APOε* and *KL-VS* were determined by SNPs analysis. Briefly, to characterize the *APOε* and the *KLOTHO* genotypes of participants, 50 ng of gDNA and two TaqMan probes were used for each assay, (rs429358 and rs7412 for *APOε* and rs9527025 and rs9536314 for *KLOTHO* (Applied Biosystems, Inc., Foster City, CA, USA).

### 4.3. Clinical Measures

FXTAS was diagnosed by an experienced clinician (RH) based on clinical and neuroimaging criteria and categorized into definite, probable, and possible FXTAS [28]. FXTAS stages were based on clinical judgment stages 0/1, subtle or questionable involvement; stage 2, definite tremor or balance problem minor interference with ADLs; stage 3, moderate tremor or balance problems and significant interference with ADLs; stage 4, severe tremor and balance problems, use of a cane or walker; stage 5, use of a wheelchair on daily basis; stage 6, bedridden, as previously reported [49].

The cognitive assessment, based on standardized testing, included the Wechsler Adult Intelligence Scale Fourth Edition (WAIS-IV) [55], was administered in 65 PM males with FXTAS on the first and subsequent visit (1–6 years apart). The presented results included VIQ, PIQ, and FSIQ. The IQ score from the first and the subsequent visits were obtained for the analysis of overtime changes and the time between visits were included as co-variant.

### 4.4. Statistical Analysis

FXTAS stage and diagnosis were compared between *APOε* and *KLOTHO* genotypes using proportional odds logistic regression models [56]. Models included *APOε* genotype (presence/absence of *APOε4* allele), *KLOTHO* genotype variants (*KL-VS^het−^* vs. *KL-VS^het+^* and or *KL-VS^hom+^*), an *APOε* by *KLOTHO* interaction effect, age, number of CGG repeats, and number of AGG interruptions. IQ (FSIQ, VIQ, PIQ) scores were compared between *APOε* genotypes using linear models that included covariates for *APOε4* allele presence/absence, *APOε2* allele presence/absence, the *APOε2*-*APOε4* interaction, age at first visit, time between visits, and IQ score at first visit, to make sure that the IQ changes were attributable to genotype rather than confounding variables. Analyses were conducted using R version 4.2.1 (23 June 2022) [57]. Proportional odds logistic regression modelling was conducted using the function polr in the R package MASS.

## Figures and Tables

**Table 1:** Participants’ demographic characteristics.

Characteristics	Overall (*n* = 245)
**Age**	
Mean (SD)	66.1 (7.93)
Median [Min, Max]	66.0 [50.0, 89.0]
**Race**	
American Indian/Alaska Native	3 (1.2%)
Asian	1 (0.4%)
More Than One Race	1 (0.4%)
Unknown	34 (13.9%)
White	206 (84.1%)
**Ethnicity**	
Hispanic or Latino	10 (4.1%)
Non-Hispanic or Latino	180 (73.5%)
Unknown	55 (22.4%)
**CGG repeats**	
Mean (SD)	86.9 (18.8)
Median [Min, Max]	84.0 [52.0, 183]
Missing	1 (0.4%)
**AGG Interruptions**	
0	122 (49.8%)
1	76 (31.0%)
2	47 (19.2%)
***APOε***	
*ε2,ε2*	3 (1.2%)
*ε2,ε3*	25 (10.2%)
*ε2,ε4*	6 (2.4%)
*ε3,ε3*	163 (66.5%)
*ε3,ε4*	47 (19.2%)
*ε4,ε4*	1 (0.4%)
***KLOTHO***	
*KL-VS^het−^*	166 (67.8%)
*KL-VS^het+^*	69 (28.2%)
*KL-^hom+^*	10 (4.1%)
**FXTAS Stage**	
0	14 (5.7%)
1	20 (8.2%)
2	44 (18.0%)
3	64 (26.1%)
4	39 (15.9%)
5	18 (7.3%)
Missing	46 (18.8%)
**FXTAS Diagnosis**	
No	25 (10.2%)
Possible	34 (13.9%)
Probable	37 (15.1%)
Definite	103 (42.0%)
Missing	46 (18.8%)

**Table 2:** Participant characteristics by FXTAS diagnosis.

	FXTAS (*n* = 165)	No FXTAS (*n* = 34)	No Diagnosis (*n* = 46)	Overall (*n* = 245)
**Age**				
Mean (SD)	66.2 (7.44)	63.3 (6.40)	67.8 (10.0)	66.1 (7.93)
Median [Min, Max]	66.0 [51.0, 85.0]	63.0 [50.0, 77.0]	68.0 [50.0, 89.0]	66.0 [50.0, 89.0]
**Race**				
American Indian/Alaska Native	2 (1.2%)	1 (2.9%)	0 (0%)	3 (1.2%)
Asian	1 (0.6%)	0 (0%)	0 (0%)	1 (0.4%)
More Than One Race	1 (0.6%)	0 (0%)	0 (0%)	1 (0.4%)
Unknown	14 (8.5%)	1 (2.9%)	19 (41.3%)	34 (13.9%)
White	147 (89.1%)	32 (94.1%)	27 (58.7%)	206 (84.1%)
**Ethnicity**				
Hispanic or Latino	7 (4.2%)	0 (0%)	3 (6.5%)	10 (4.1%)
Non-Hispanic or Latino	127 (77.0%)	29 (85.3%)	24 (52.2%)	180 (73.5%)
Unknown	31 (18.8%)	5 (14.7%)	19 (41.3%)	55 (22.4%)
**CGG**				
Mean (SD)	89.4 (18.6)	77.5 (17.0)	84.8 (18.4)	86.9 (18.8)
Median [Min, Max]	87.0 [52.0, 183]	76.0 [53.0, 135]	83.0 [53.0, 130]	84.0 [52.0, 183]
Missing	0 (0%)	1 (2.9%)	0 (0%)	1 (0.4%)
**AGG Interruptions**				
0	83 (50.3%)	20 (57.1%)	19 (42.2%)	122 (49.8%)
1	50 (30.3%)	11 (31.4%)	15 (33.3%)	76 (31.0%)
2	32 (19.4%)	4 (11.4%)	11 (24.4%)	47 (19.2%)
***APOε*** **Genotype**				
*ε2,ε2*	3 (1.8%)	0 (0%)	0 (0%)	3 (1.2%)
*ε2,ε3*	17 (10.3%)	5 (14.3%)	3 (6.7%)	25 (10.2%)
*ε2,ε4*	5 (3.0%)	0 (0%)	1 (2.2%)	6 (2.4%)
*ε3,ε3*	111 (67.3%)	24 (68.6%)	28 (62.2%)	163 (66.5%)
*ε3,ε4*	29 (17.6%)	6 (17.1%)	12 (26.7%)	47 (19.2%)
*ε4,ε4*	0 (0%)	0 (0%)	1 (2.2%)	1 (0.4%)
***KLOTHO*** **Genotype**				
*KL-VS^het−^*	110 (66.7%)	25 (71.4%)	31 (68.9%)	166 (67.8%)
*KL-VS^het+^*	50 (30.3%)	10 (28.6%)	9 (20.0%)	69 (28.2%)
*KL-^hom+^*	5 (3.0%)	0 (0%)	5 (11.1%)	10 (4.1%)

**Table 3:** Comparison of FXTAS stage between *KLOTHO* genotypes, by *APOε4* genotypes, and between *APOε4* genotypes, by *KLOTHO* genotype, from proportional odds logistic regression analysis.

***APOε4* Genotype**	**Comparison**	**Odds Ratio (95% CI)**	***p*** **-Value**
No	(*KL-VS^het+^* + or *KL-VS^hom+^*) − (*KL-VS^het−^*)	1.123 (0.612, 2.062)	0.707
Yes	(*KL-VS^het+^* + or *KL-VS^hom+^*) − (*KL-VS^het−^*)	1.63 (0.348, 7.644)	0.535
***KLOTHO* Genotype**	**Comparison**	**Odds Ratio (95% CI)**	***p*** **-Value**
*KL-VS^het−^*	*APOε4* allele present—No *APOε4* allele	1.396 (0.644, 3.027)	0.398
*KL-VS^het+^* + or *KL-VS^hom+^*	*APOε4* allele present—No *APOε4* allele	2.026 (0.46, 8.92)	0.351

Odds ratio is ratio of odds of higher vs. lower FXTAS stage.

**Table 4:** Comparison of FXTAS diagnosis between *KLOTHO* genotypes, by *APOε4* genotype, and between *APOε4* genotypes, by *KLOTHO* genotype.

***APOε4* Genotype**	**Comparison**	**Odds Ratio (95% CI)**	***p*** **-Value**
No	(*KL-VS^het+^* + or *KL-VS^hom+^*) − (*KL-VS^het−^*)	1.24 (0.616, 2.496)	0.547
Yes	(*KL-VS^het+^* + or *KL-VS^hom+^*) − (*KL-VS^het−^*)	1.077 (0.107, 10.854)	0.950
***KLOTHO* Genotype**	**Comparison**	**Odds Ratio (95% CI)**	***p*** **-Value**
*KL-VS^het−^*	*APOε4* allele present—No *APOε4* allele	1.239 (0.502, 3.058)	0.642
*KL-VS^het+^* + or *KL-VS^hom+^*	*APOε4* allele present—No *APOε4* allele	1.076 (0.112, 10.318)	0.949

Odds ratio is ratio of odds of higher vs. lower FXTAS diagnosis.

**Table 5:** Comparison of FXTAS stage between *APOε2*, by *APOε4* status, and between *APOε4*, by *APOε2* status.

***APOε4***	**Comparison**	**Odds Ratio** **(95% CI)**	***p*** **-Value**
No	*APOε2* allele present—No *APOε2* allele	0.727 (0.311, 1.698)	0.461
Yes	*APOε2* allele present—No *APOε2* allele	0.758 (0.149, 3.855)	0.738
***APOε2***	**Comparison**	**Odds Ratio** **(95% CI)**	***p*** **-Value**
No	*APOε4* allele present—No *APOε4* allele	1.477 (0.702, 3.105)	0.304
Yes	*APOε4* allele present—No *APOε4* allele	1.539 (0.288, 8.22)	0.614

Odds ratio is ratio of odds of higher vs. lower FXTAS stage.

**Table 6:** Comparison of FXTAS diagnosis between *APOε2*, by *APOε4* status, and between *APOε4*, by *APOε2* status.

***APOε4***	**Comparison**	**Odds Ratio** **(95% CI)**	***p*** **-Value**
No	*APOε2* allele present—No *APOε2* allele	0.539 (0.218, 1.329)	0.179
Yes	*APOε2* allele present—No *APOε2* allele	0.61 (0.097, 3.822)	0.597
***APOε2***	**Comparison**	**Odds Ratio** **(95% CI)**	***p*** **-Value**
No	*APOε4* allele present—No *APOε4* allele	1.142 (0.451, 2.891)	0.779
Yes	*APOε4* allele present—No *APOε4* allele	1.292 (0.206, 8.118)	0.785

Odds ratio is ratio of odds of higher vs. lower FXTAS diagnosis.

**Table 7:** Comparison of change in verbal IQ between *APOε2*, by *APOε4* status, and between *APOε4*, by *APOε2* status between FXTAS subjects (*n* = 65).

***APOε4***	**Comparison**	**Difference in Means** **(95% CI)**	***p*** **-Value**
No	*APOε2* allele present—No *APOε2* allele	8.4 (−11, 181)	0.071
Yes	*APOε2* allele present—No *APOε2* allele	−4.6 (−181, 91)	0.496
***APOε2***	**Comparison**	**Difference in Means** **(95% CI)**	***p*** **-Value**
No	*APOε4* allele present—No *APOε4* allele	3.7 (−51, 121)	0.399
Yes	*APOε4* allele present—No *APOε4* allele	−9.3 (−231, 51)	0.182

Model includes *APOε2* and *ε4* alleles (presence/absence), their interaction, time between visits, age at first visit, and verbal IQ at first visit.

## Data Availability

The data that support the findings of this study are available from the corresponding author (F.T.), upon reasonable request.

## Supplementary Materials

The following supporting information can be downloaded at: https://www.mdpi.com/article/10.3390/ijms25158103/s1.
