# Supplementary material for: Apolipoproteine and KLOTHO Gene Variants Do Not Affect the Penetrance of Fragile X-Associated Tremor/Ataxia Syndrome
Source: Int J Mol Sci. 2024 Jul 25;25(15):8103. doi: 10.3390/ijms25158103 (PMC11312271; doi:10.3390/ijms25158103)
Supplement: Supplementary file 1 [file ijms-25-08103-s001.zip › Figures S1-S4 Supplementary_Winarni et al.pdf]

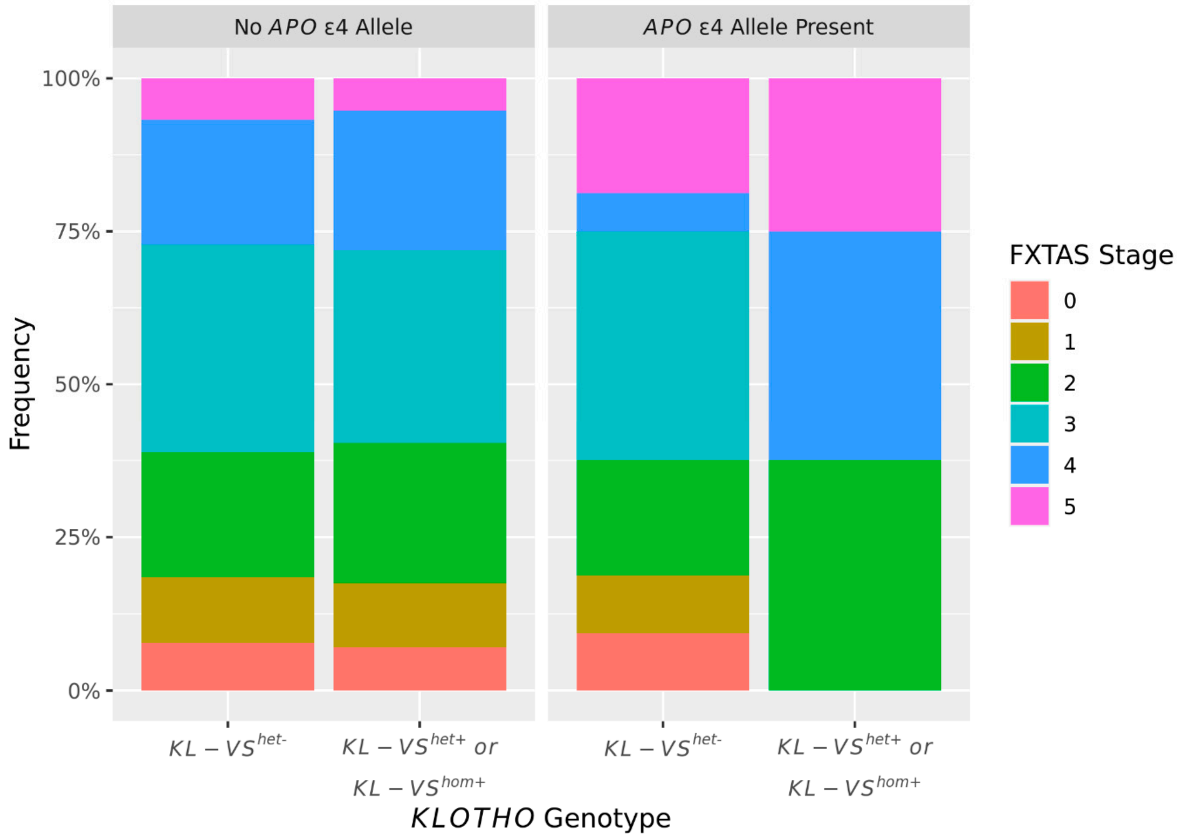

Figure S1. Stacked Barplot of FXTAS Stage by *APOε4* and *KLOTHO Variants*

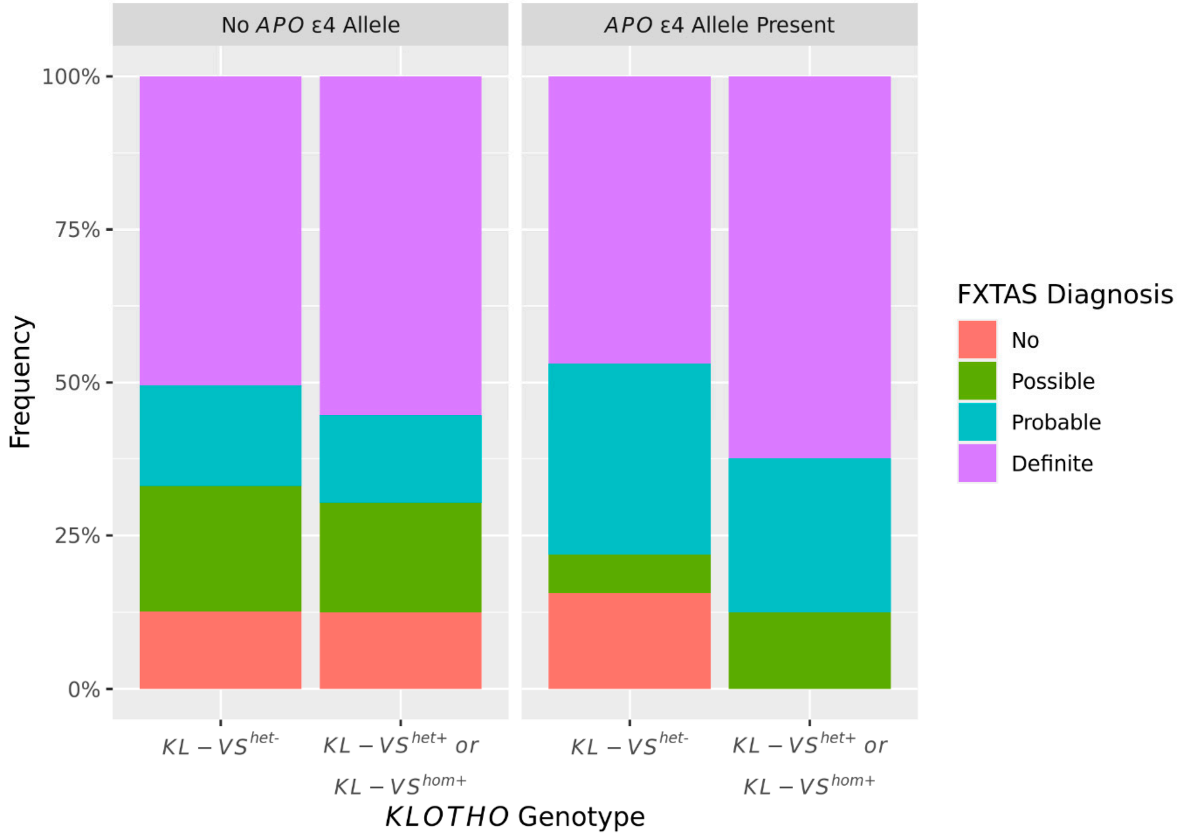

Figure S2. Barplot of FXTAS Diagnosis by *APOε4* and *KLOTHO variants*

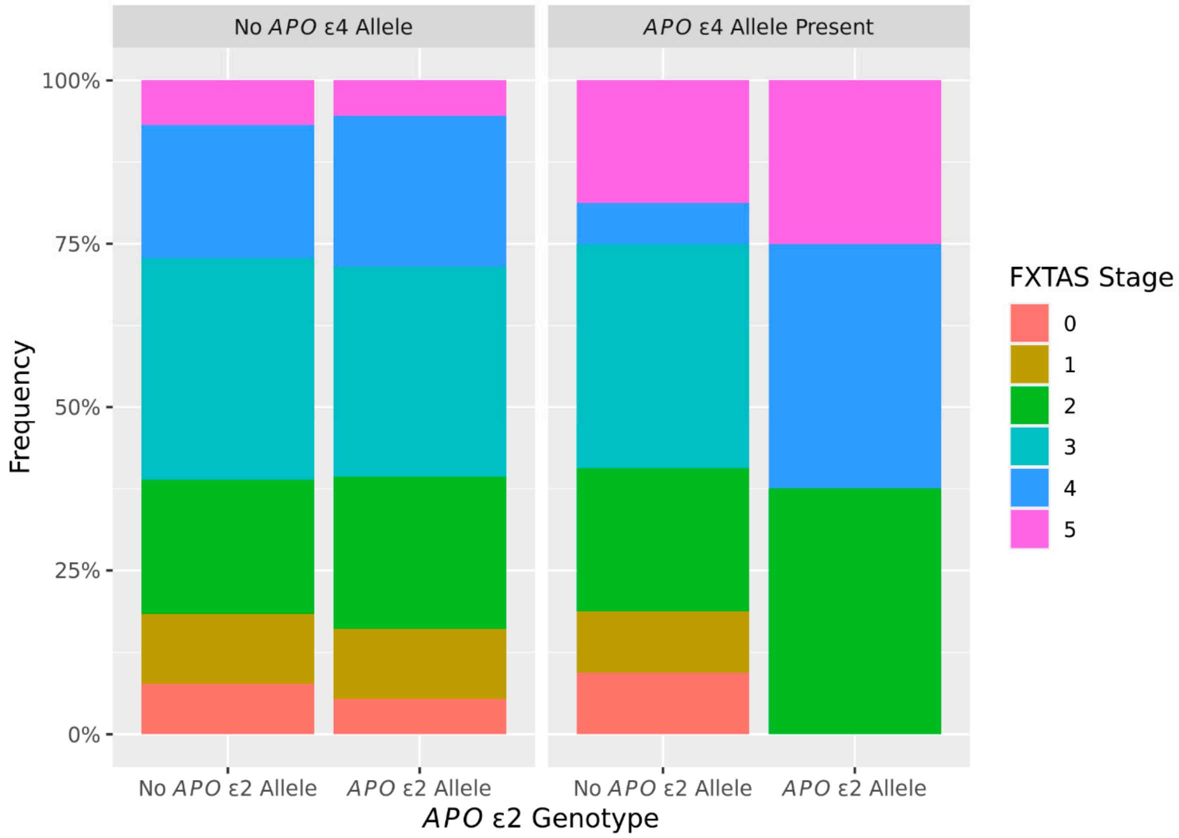

Figure S3. Barplot of FXTAS Stage by *APOε2* and *APOε4 Genotype*

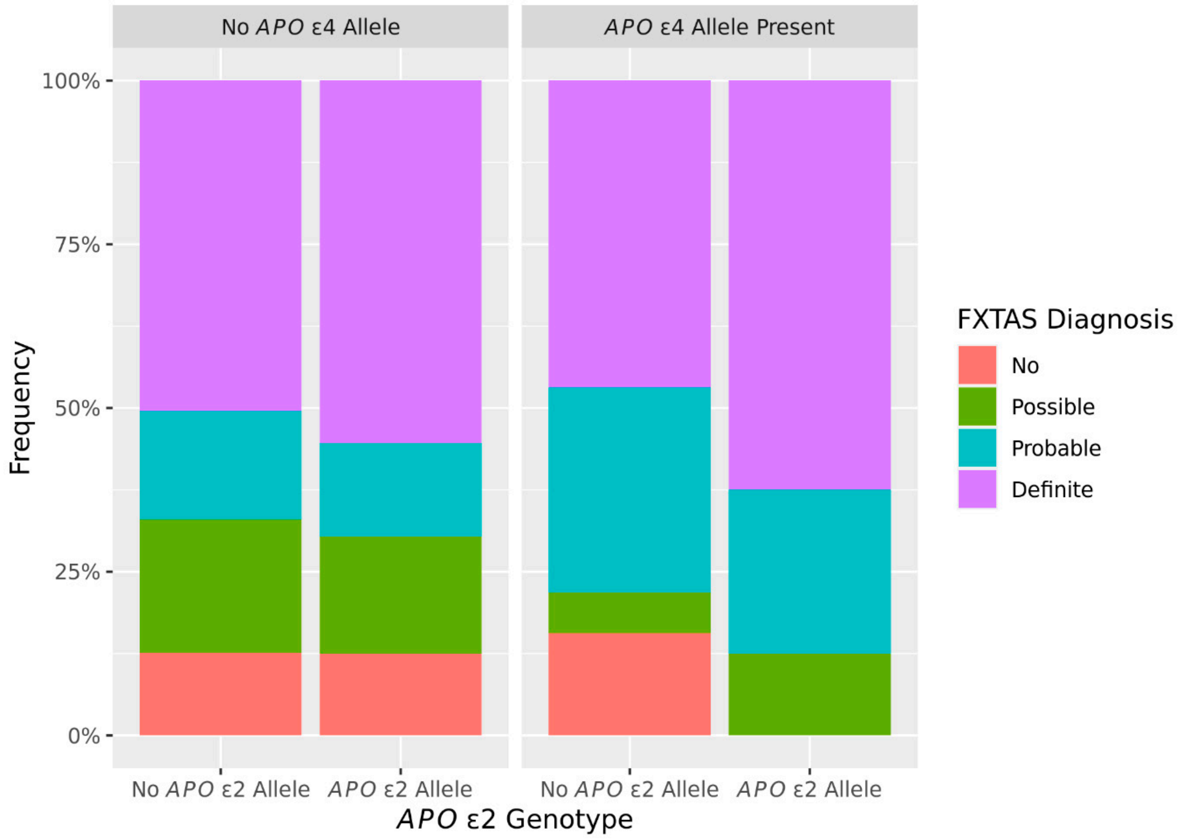

Figure S4. Barplot of FXTAS Diagnosis by *APOε2* and *APOε4 Genotype*
